# Supplementary material for: The Effects of an Order-Assist Mobile Application on Pediatric Anesthesia Safety: An Observational Study
Source: Children (Basel). 2023 Nov 27;10(12):1860. doi: 10.3390/children10121860 (PMC10741693; doi:10.3390/children10121860)
Supplement: Supplementary file 1 [file children-10-01860-s001.zip › children-2706068-supplementary.pdf]

There are 15 questions in total in this survey.

Please circle the responses that apply to you.

1. How many years have you worked as an anesthesia nurse?

   ① Less than 3 years
   ② Equal or more than 3 years but less than 5 years
   ③ Equal or more than 5 years but less than 10 years
   ④ Equal or more than 10 years

2. When preparing for a pediatric anesthesia, how often do you expect to modify an existing verbal order?

   ① No changes
   ② Expect one or two orders per anesthesia
   ③ More than 3 orders per anesthesia
   ④ Constant changes

3. When you receive conventional verbal order, how many times during the preparation of pediatric anesthesia do you have to double-check with the anesthesiologist due to unclear, incorrect, or missing orders?

   ① More than one times per anesthesia
   ② Once per every two anesthesia
   ③ Once per 3 or 4 anesthesia.
   ④ Once per equal or more than 5 anesthesia
   ⑤ Never

4. How much time do you need to prepare for a pediatric anesthesia?

① Less than 5 minutes
② Equal or more than 5 minutes but less than 10 minutes
③ Equal or more than 10 minutes but less than 15 minutes
④ Equal or more than 15 minutes but less than 20 minutes
⑤ Equal or more than 20 minutes

5. How often do you leave the operating room during a round of anesthesia because of a change or new order from the anesthesiologist?

① Never
② Once
③ Twice
④ Equal or more than3 times

6. How long do you leave the operation room during each round of anesthesia due to changes or additions to the anesthesiologist's order?

① Less than 5 minutes
② Equal or more than 5 minutes but less than 10 minutes
③ Equal or more than 10 minutes but less than 15 minutes
④ Equal or more than 15 minutes but less than 20 minutes
⑤ Equal or more than 20 minutes

** Medicine

Ketamine : 4.8 mg

Mobinul : 0.02 mg (0.1 ample)

Remifentanil : 0.25mg in 5DS 50cc

Dexmedetomidine : 200mcg in 50cc

Esmeron : 4 mg

Sugammadex : 10 mg

** Equipments

Laryngoscope :
UEscope #0 Miller(Straight)

ET tube type : Cuffed

ET tube size : #3.5

Temperature probe : esophageal probe

Air Warming Blanket

** iv line & fluid

main fluid : maintain current fluid

fluid 2 : 5% albumin ( 20% albumin : 5DS = 1:3 )

< Additional orders >

Prepare ABGA

7. The content in the box on the left is an example of a pediatric anesthesia preparation order sent to a nurse on her mobile phone via the pediatric anesthesia assistant app. Have you ever received an order from a doctor that looks like the one on the left?

① Yes, I have.
② No, I have not.

8. Compared to traditional verbal orders, do you find it more convenient to have them delivered to your phone via the app, as shown on the left, to prepare a pediatric anesthesia?

① Yes, I do.
② No difference.
③ No, I don’t. It is inconvenient.

9. Compared to traditional verbal orders, do you find it more effective to have them delivered to your phone via the app, as shown on the left, to reduce medication errors due to miscommunication?

① Yes, I do.
② No difference.
③ No, I don’t. It is more error prone.

10. When preparing for a pediatric anesthesia, how often do you expect to modify orders by an anaesthesiologist when you receive orders by the app?

① No Experience with the app
② No order change or adding when using the app
③ One or two order changes or adding orders when using the app
④ Three or more order changes or adding orders when using the app
⑤ Constant changes or adding orders when using the app

11. When you received app generated order, how many times during the preparation of pediatric anesthesia do you have to double-check with the anesthesiologist due to unclear, incorrect, or missing orders?

① No experience with the app
② Be sure to ask once.
③ Asked once If anesthesia is given twice.
④ Asked once after the 3rd or 4th time of anesthesia.
⑤ asked once If the number of anesthesia exceeds 5 times,
⑥ There is no case of double-check.

12. Do you think the use of the Pediatric Anesthesia Assistant app could reduce the number of times a nurse leaves the operating room to go to the prep room during anesthesia?

① Yes.
② No.

13. Do you think the use of the Pediatric Anesthesia Assistant app could reduce the amount of time nurses leave the OR to go to the prep room during anesthesia?

① Yes.
② No.

14. Please select the level of convenience you have when preparing a pediatric anesthesia order.

Note) ① ② ③ ④ ⑤ has the following meanings

① Very inconvenient
② Inconvenient
③ Average
④ Convenient
⑤ Very Convenient

1. App ( ① ② ③ ④ ⑤ )
2. EMR ( ① ② ③ ④ ⑤ )
3. Conventional verbal ( ① ② ③ ④ ⑤ )

15. Please select the likelihood of errors when preparing a pediatric anesthesia using each method.

Note) ① ② ③ ④ ⑤ has the following meanings

① More errors
② Many errors
③ Moderate
④ Few errors
⑤ Fewer errors

1. App ( ① ② ③ ④ ⑤ )
2. EMR ( ① ② ③ ④ ⑤ )
3. Conventional verbal ( ① ② ③ ④ ⑤ )

**This is the last questionnaire. Thank you for your participation.**
